# How Our Cognition Shapes and Is Shaped by Technology: A Common Framework for Understanding Human Tool-Use Interactions in the Past, Present, and Future

**DOI:** 10.3389/fpsyg.2018.00293

**Published:** 2018-03-07

**Authors:** François Osiurak, Jordan Navarro, Emanuelle Reynaud

**Affiliations:** ^1^Laboratoire d’Etude des Mécanismes Cognitifs (EA 3082), Institut de Psychologie, Université de Lyon, Lyon, France; ^2^Institut Universitaire de France, Paris, France

**Keywords:** tool use, technology, brain–computer interface, automation, technical reasoning

## Abstract

Over the evolution, humans have constantly developed and improved their technologies. This evolution began with the use of physical tools, those tools that increase our sensorimotor abilities (e.g., first stone tools, modern knives, hammers, pencils). Although we still use some of these tools, we also employ in daily life more sophisticated tools for which we do not systematically understand the underlying physical principles (e.g., computers, cars). Current research is also turned toward the development of brain–computer interfaces directly linking our brain activity to machines (i.e., symbiotic tools). The ultimate goal of research on this topic is to identify the key cognitive processes involved in these different modes of interaction. As a primary step to fulfill this goal, we offer a first attempt at a common framework, based on the idea that humans shape technologies, which also shape us in return. The framework proposed is organized into three levels, describing how we interact when using physical (Past), sophisticated (Present), and symbiotic (Future) technologies. Here we emphasize the role played by technical reasoning and practical reasoning, two key cognitive processes that could nevertheless be progressively suppressed by the proficient use of sophisticated and symbiotic tools. We hope that this framework will provide a common ground for researchers interested in the cognitive basis of human tool-use interactions, from paleoanthropology to neuroergonomics.

## Introduction

Have you already wondered how researchers living 70 years ago could contact an editor to know whether their manuscript was still under review or not after 5 months? They certainly had to write a mail and wait for a response, perhaps 5 weeks after. Nowadays, we send emails and expect an answer by 2 or 3 days. Perhaps in 1000 years, researchers will just have to think of this and they will receive the answer instantly. These different modes of interaction illustrate the constant modification of our technologies over time, a phenomenon that characterizes our species (Boyd and Richerson, 1985). The ultimate goal of research on this topic is to identify the key cognitive processes involved in these different modes of interaction. As a primary step to fulfill this goal, we offer a first attempt at a common framework, based on the idea that humans shape technologies, which also shape us in return.

**FIGURE 1:**
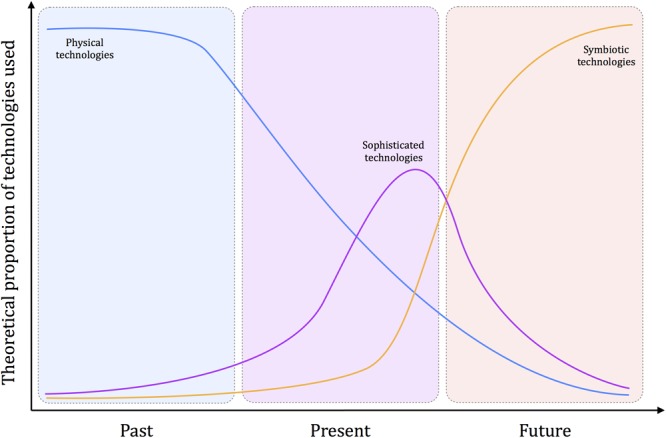
Theoretical proportion of physical, sophisticated, and symbiotic technologies used over time. The core idea is that, at the species level, physical technologies are anterior to sophisticated technologies, which are also anterior to symbiotic technologies. Over time, physical technologies (e.g., stone tools, knifes, hammers) tend to decrease and could be completely absent in a far future. Sophisticated technologies have appeared later and are now a great part of the technologies we use (i.e., interface-based technologies). Again, it can be hypothesized that this kind of technologies will be less and less used. Finally, symbiotic technologies are developing now even if they remain rarely used (e.g., brain–computer interfaces). In a far future, it can be thought that humans will profusely and uniquely use these technologies. The three colored panels correspond to the three time periods (Past, Present, and Future). The color associated to each kind of technologies corresponds to the color of the period where a given technology is dominant (Past: the reign of physical technologies; Present: the reign of sophisticated technologies; Future: the reign of symbiotic technologies).

**FIGURE 2:**
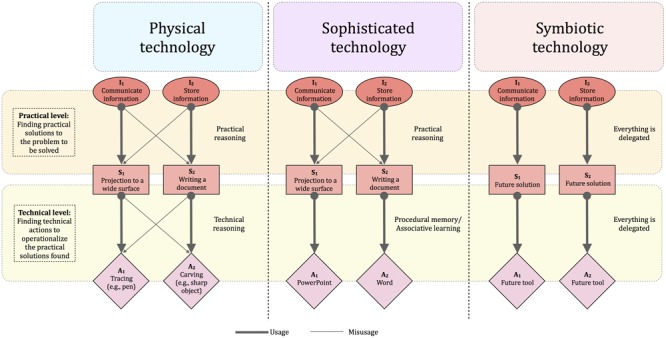
Neurocognitive processes involved in physical, sophisticated, and symbiotic technologies. The core idea is that humans develop technologies in order to satisfy intentions (I). To do so, they have to select appropriate practical solution (S), leading then to the selection and application of technical actions (A). In the case of physical technologies, the intention can be to communicate information (I_1_). This can be achieved either by projecting information to a wide surface (S_1_) or writing a document (S_2_). There is no bijection between the “domain” of intentions and the “domain” of practical solutions in that a given intention can be achieved through two different practical solutions and, inversely, the same practical solutions can be useful to achieve a given intention. At this practical level, humans have to imagine the most appropriate practical solution. Then, once a given practical solution selected (e.g., S_1_), it has to been operationalized by selecting and applying a technical action (e.g., A_1_). For instance, if the practical solution is to project information to a wide surface, the technical solution can be tracing by using a pencil on a wall. Again, there is no bijection between the “domain” of practical solutions and the “domain” of technical actions. For physical technologies, humans have to do technical reasoning to select and apply the appropriate technical actions. However, for sophisticated technologies, this technical level is suppressed, people having just to learn the procedure thought by the maker/designer to interact with the technology (e.g., pressing a button to activate a given function). Interestingly, for both physical and sophisticated technologies, people are still free to reason at a practical level in order to select which practical solutions to choose. For symbiotic technologies, this practical level is suppressed, with the idea that the intention is directly implemented, without having to decide between different practical solutions and, as a result, technical actions. Bold and thin lines represent, respectively, usages and misusages, that is, the usual or unusual path a user can follow to satisfy an intention. Sophisticated technologies tend to suppress misusages at a technical level, because people have no other possibilities than pressing buttons, for instance, to power PowerPoint. However, they can still divert the pre-established use of PowerPoint (i.e., communication device) in order to fulfill another intention (i.e., external memory). For symbiotic tools, both technical reasoning and practical reasoning from the user could be suppressed, because the user intervenes neither at the technical level, nor at the practical level.

The framework proposed is organized into three levels, describing how we interact when using physical (Past), sophisticated (Present), and symbiotic (Future) technologies^1^. The temporal gradient introduced here implies that, at the species level, physical technologies are anterior to sophisticated ones, which are anterior to symbiotic ones, so that the theoretical proportion of use for each kind of technology is supposed to evolve over time (**Figure 1**). The distinction made between these different kinds of technology is also theorized here at a cognitive level, based on the idea that our modifications on the world are first guided by an intention, needing then the selection of a practical solution (i.e., the practical level), and finally the selection and application of a technical action (i.e., the technical level; **Figure 2**). The thesis defended here is that the technical evolution from physical to sophisticated and symbiotic technologies tends to progressively suppress the technical and practical levels.

Three caveats need to be made at this point. First, there is no overview in the literature about the cognitive processes involved in the different interactions we have with tools and technologies. The major reason for this lack is that this requires a critical, epistemological development as to the way of organizing the field so that researchers from different topics (e.g., stone tools, brain–computer interaction) could communicate within a single and comprehensive framework. The goal of this paper is to fill this gap, by attempting to provide a structured way of organizing the literature based on the evolution of our technology over time. This attempt could be a good starting point for developing such a framework in the future. Second, many cognitive processes are involved in our interactions with tools and technologies. Here we could not address all of them and preferred to concentrate our attention on two key cognitive processes, namely, technical reasoning and practical reasoning. Of course, further theoretical development would be needed to complete our analysis. Third, as with other humans, our interactions with tools and technologies can take different forms according to the role taken by technology (e.g., competition, collaboration). These different levels of interaction that most directly deal with the “social” aspect will be addressed partly in this paper, particularly in the third section. Nevertheless, we acknowledge that a more comprehensive review based on this level of analysis could complete the present review, discussing the potential parallel between our interactions with social (e.g., humans) and non-social (e.g., technologies) agents.

## The Past: Physical Tools

Physical tools can be defined as those tools that increase our sensorimotor abilities (Virgo et al., 2017). Although we still use a wide variety of physical tools (e.g., hammer, knife), it can be considered that they correspond to the first tools humans have made and used in *pre*-history. At a cognitive level, the use of all physical tools shares the need for the user to understand physical principles (e.g., percussion, cutting). The characteristics of early stone tools indicate that makers showed evidence of a basic understanding of stone fracture mechanics (Hovers, 2012). The use of physical tools by modern humans also requires this form of physical understanding (Bril et al., 2010).

Some patients can meet difficulties to use everyday tools after left brain damage (Osiurak and Rossetti, 2017). The difficulties concern not only the selection of the appropriate tool, but also the mechanical action performed (e.g., pounding a nail by rubbing it on the nail instead of hammering with it). The same difficulties can be observed when they are asked to solve mechanical problems by using novel tools (Goldenberg and Hagmann, 1998; Jarry et al., 2013). Taken together, these findings indicate that the use of physical tools is grounded on the ability to reason about physical properties of tools and objects based on mechanical knowledge. This is what we call “technical reasoning” (Osiurak et al., 2010; Osiurak and Badets, 2016). This reasoning is critical to form a mental representation of the mechanical action intended. It is also the key process allowing us to generate instances of “technical misusage” (**Figure 2**) also called “function creep,” corresponding to the use of a tool in an unusual way (Osiurak et al., 2009). Such instances can be observed relatively early in humans. A 2-years-old child can, for instance, use a tea spoon to hammer a piece of cheese in his mashed carrots, calling the spoon “a hammer.” This child knows that the spoon is not a hammer but finds funny to hammer the cheese and handy to use the spoon to do so at that time.

Technical reasoning could be unique to humans (e.g., Penn et al., 2008), explaining a certain number of our specificities such as the use of one tool to create another (e.g., stone knapping) or the use of complex tools that transform our motor energy into different mechanical energies (Osiurak, 2017). Convergent evidence from neuropsychology and cognitive neuroscience indicates that technical reasoning could engage the area PF within the left inferior parietal cortex (Goldenberg and Spatt, 2009; Reynaud et al., 2016), which does not in macaques and other non-human primates (Orban and Caruana, 2014).

Before going on to the next section, one important aspect needs to be considered. Technical reasoning is critical for the making of any technology (physical, sophisticated, symbiotic). For physical technologies, there is no real distance between the maker and the user in that the user needs to mentally make the technology before the use (Osiurak and Heinke, 2017). If you intend to cut a tomato, you are free to select a wide variety of tools. Nevertheless, your selection is based on the physical properties of the tomato, leading you to choose a tool with the appropriate physical properties relatively to the tomato. In a way, you first make your tool mentally (e.g., thinking about something sharp and solid enough) and then you select it *really* accordingly. Things are different for sophisticated technologies, which mainly correspond to interface-based technologies (e.g., computers). A key characteristic of these technologies is that the maker/designer has facilitated the interaction, so that the user has no longer to understand the physical principles underlying the use. In this case, the user does not make mentally the tool before the use but learn the arbitrary relationship between the motor response and its effect. The corollary is that sophisticated technologies may not require, at the technical level (**Figure 2**), technical reasoning skills, but more basic cognitive processes such as associative learning and procedural memory (Osiurak and Heinke, 2017). At least two lines of evidence support this view. First, interface-based technologies (e.g., touchscreens) can be easily used by infants, despite moderate skills to use physical tools (Beck et al., 2011). Likewise, many non-human animals including tool users (e.g., baboons) can use touchscreens very quickly in the absence of any signs of physical tool use (Claidière et al., 2014). Second, patients with damage to the left inferior parietal cortex are impaired to use physical tools, but not interface-based technologies. The opposite pattern can be observed in patients with deficits of procedural memory (e.g., Parkinson’s disease), indicating a double dissociation between the ability to use physical versus sophisticated technologies (see Osiurak, 2014, 2017).

## The Present: Sophisticated Tools

Stopping the alarm clock after waking up, using tramways, driving a car, interacting with a smartphone, taking the elevator, and so on. With the sophistication of tools and the advent of cognitive tools (e.g., computer spreadsheet) the distance between the making and the use has dramatically increased, so we use many tools we could never build in a lifetime. This does not change the way we interact with tools: the purpose of a tool is not in the tool itself, but in the user’s intentions. A computer screen can be used to stick notes, as a visual barrier, as a mirror, and so forth (i.e., technical misusage). This fact remains whatever the nature of the tool considered, from a very simple stone tool to the most advanced smartphone (e.g., reflecting sunlight). There is a limit, however, in the lack of freedom offered by sophisticated tools to its users at the technical level, because the use of these tools for their usual function needs to master pre-established procedures (see above).

Some sophisticated tools, often referred as automation, do not tend to extend humans but rather to replace them (Young et al., 2007). Those tools that replaces us tend to be poorly accepted by individuals (Navarro et al., 2011). The design of these tools also questions about the human role in our societies, and about what should be automated or not (Hancock, 2014). For instance, a highly automated task completion is often considered as dehumanizing (Coeckelbergh, 2015). People also select an automatic completion of the task only if much more effective than a manual completion (Osiurak et al., 2013; Navarro and Osiurak, 2015, 2017), as if humans tend to avoid the loss of freedom associated to sophisticated tools (**Figure 2**).

Tool use is not neutral for users. Of course, tools are changing the way humans do things, but tools also change humans themselves (Hancock, 2007). All the data available on the Internet provide considerable benefits, yielding information easily. But, it also alters the way people memorize information itself in favor of a recall of where to access it Sparrow et al. (2011). Is it for the best or for the worst? This is not a new question, at least in the cognitive ergonomics field. Parasuraman and Riley (1997) stated that automation “*changes the nature of the work that humans do, often in ways unintended and unanticipated by the designers of automation*” (p. 231). Use is described here as the human proneness to activate automation when available. Besides a correct use of automation, misuse (i.e., overreliance on automation) and disuse (i.e., underutilization of automation) have been reported. Thus, the human is reasoning about its interactions with sophisticated tools to adjust his/her behavior according to the context and his/her own objectives (Leplat, 1990). For instance, automation use was found to be related to a balance between trust in automation and user self-confidence (Lee and Moray, 1994). These data can be interpreted as the human nature to keep reasoning based on internal and external assessments (i.e., practical reasoning). This is what we refer to as practical misusage, that is, the ability to divert the pre-established use of a tool (e.g., PowerPoint as a communication device) to fulfill another intention (e.g., storing information; **Figure 2**). A research issue to investigate is the neural bases that support this “practical reasoning.” Are there (a) partly the same as those required by technical reasoning? (b) Rather common to those associated to logical reasoning? Or (c) implying areas known to be engaged in interactions with other humans that would be recycled to reason on human–machine interactions?

Another aspect specific to sophisticated tools is that the perception or inference of tool functions could be sometimes complicated because of the distance between the maker and the user, favoring the occurrence of inappropriate and ineffective use. To counter this phenomenon, a human-centered design has been proposed (Billings, 1991). This design process widely used in a variety of domains (François et al., 2016) is based on the rationale that tool designers should take into account as much as possible users’ logic and characteristics during the tool design process. In a way, the consideration of the user in the design process aims at reducing the distance between the maker and the user. Nevertheless, if we assume that humans are keen on practical reasoning, this quest is necessary deceptive as there is no universal reasoning process and, thus, neither universal human–tool interaction, nor natural interaction with sophisticated tools. Inversely, the human–tool interaction is rather artificial because based on an artifice (i.e., a sophisticated tool) for which the user ignores, at least part of, the design philosophy and the working principle.

## The Future: Symbiotic Tools

**Kid #1**: “*You mean you have to use your hands?*”

**Kid #2**: “*That’s like a baby’s toy!*”

—*Back to the Future Part II*

Predicting the future of our technology could be a fortune teller’s job, had there not been a few mesmerizing anticipation movies and books, featuring great inventions feeding from contemporary science, the society’s aspirations, and feeding back companies striving for developing them: inventions such as the Blade Runner flying autonomous cars or the gesture-based user interface from Minority Report prefigure the tools of the future. Some may never be created, some may be part of our everyday lives in 30 years, as the video calls from the first Blade Runner movie are part of our modern lives. This sneak peek into the future shows that all these tools have one thing in common: they seem to be operated seamlessly and conveniently by the user, reducing or abolishing four main constraints: mechanics, space, time, and effort (Osiurak, 2014). Although the depicted vision of our future world is always more technology-oriented, machines never overwhelm the user, who is becoming a part of a human–machine system, as the “commander-in-chief.”

Most of the promised futuristic and fantastic tools are operated by thought, voice, or gestures. Because human–machine interaction through devices such as a mouse or keyboard is slow, inefficient, and sometimes not even feasible, the possibility of communicating with machines directly from our thoughts has emerged (Schalk, 2008). The brain–computer interface (BCI) (Wolpaw et al., 2002) field has then rapidly gained interest, first because it could be used in motor rehabilitation programs (Chaudhary et al., 2016), as the aim of BCI is to translate brain activity (“thoughts”) into commands understandable by a machine. For achieving this, brain activity is captured by the means of sensors, pre-treated, and assigned to a corresponding action to be performed by the artificial system through an adaptive algorithm that learns to discriminate classes in the brain signals recorded (Mitchell, 1997; Bishop, 2006). A successful BCI interaction very often includes a learning phase attuning the technology to the specificity of the user’s cognitive system. The structural inter-individual heterogeneity of the brains themselves, the functional differences, even the intra-individual differences from a time to another, will push the need for the learning algorithms to be highly adapted to a particular individual, if not to his particular mood.

Following this, the tantalizing promises of body-and-mind-operated tools, responding efficiently to the user’s intentions, come with the need of individualizing the technology operating the machine. Brain–machine communication needs to be truly adapted to each specific individual for brain patterns to be successfully converted into thoughts. In this ultra-individualized technology, the individual and the tool will then form a system in a tight relationship, depending on each other to “perform” appropriately. The tool is then embodied within the user, and the system they form could be designated as a “symbiotic tool” (Licklider, 1960; Brangier and Hammes-Adelé, 2011). Within this tight interaction, the human has the intention, then the tool operates the technical and practical choices (i.e., suppression of the technical and practical levels; **Figure 2**).

On the journey to a Future in which Technology and Man form a symbiotic system, a few issues remain to be addressed. The first one is the acceptation issue (Davis, 1989). Are we designed to pair with synthetic devices? Can we and shall we accept to be part of a man–machine system? Tools of the Present need the user to accept them. We postulate that the future symbiotic tools will need the user to incorporate them. The second point is to explore the limits of the human cognitive system in terms of BCI performance. To function as smoothly and perfectly as in the Avatar movie for example, many technical issues have to be solved from the maker: the sensors need to be implanted, miniaturized; the algorithms need to be fast and reliable, etc. (Lebedev and Nicolelis, 2006). If the machine-related issues will without any doubt be resolved at some point, only few researches have tackled the man-related issue. Are the neural signals encoding our thoughts specific and reliable enough to be translated into a crystal-clear command? For how long can we maintain a neural state corresponding to a sustained command? Are we (all) designed to be good BCI-commanders, and always? Studies on BCI illiteracy show that 20% of the population cannot produce the brain patterns required for a BCI system to function properly (Vidaurre and Blankertz, 2010). Are their brains faulty, or the techniques immature?

These questions relate to the fundamental enigma of the cognitive system: how can our complex thoughts, dreams, feelings, creativity, instinct, etc. be encoded into less than 10^15^ signals? How can an infinite and unexplored mental world be created by a finite and defined material support? The birth of neuroergonomics (Hancock and Szalma, 2003; Parasuraman, 2003) will certainly help to start answering these issues, and to develop efficient channels of communication with technology.

## Conclusion

In this review, we depict the different cognitive modes of interaction we have with physical, sophisticated and symbiotic tools. The key idea is that there could be a trend to progressively suppress our involvement at technical and practical levels (**Figure 2**). Interestingly, when considering symbiotic tools, users might be, a day, restricted to produce only intentions and will delegate all remaining efforts and choices to machines. The key issue is whether this restriction has to be viewed as a source of freedom or not? After all, should this scenario be true, what will humans do to occupy their available brain time? We are also aware that this review is biased by our ability to envision future tools, and how technology will evolve in a far future. Perhaps our conception of symbiotic tools is limited, considering only tools that transform our conscious intentions into responses. However, perhaps we will be able to develop technologies that will produce responses based on unconscious thoughts, thereby anticipating our needs even if we are unable to correctly generate them – or even before we generate them (e.g., sending an email to an editor before we intend to do so). In this respect, a critical question for future research is to determine whether our technological cultural evolution will reach an asymptote as suggested here, or whether other forms of technological interactions will emerge in a far future, again shaping our cognition in return.

## Author Contributions

All authors listed have made a substantial, direct and intellectual contribution to the work, and approved it for publication.

## Conflict of Interest Statement

The authors declare that the research was conducted in the absence of any commercial or financial relationships that could be construed as a potential conflict of interest.

## Footnotes

1 The terms tool and technology will be hereafter used interchangeably and in a broad sense to refer to any environmental object useful to increase the user’s sensorimotor or cognitive capacities (Osiurak et al., 2010).
